# Attitudes and knowledge about direct and indirect risks among conventional and complementary health care providers in cancer care

**DOI:** 10.1186/s12906-018-2106-z

**Published:** 2018-01-31

**Authors:** Trine Stub, Sara A. Quandt, Thomas A. Arcury, Joanne C. Sandberg, Agnete E. Kristoffersen

**Affiliations:** 1 Wake Forest School of Medicine, Department of Epidemiology and Prevention, Division of Public Health Sciences, Medical Center Boulevard, Winston-Salem, NC 27157 USA; 2 Wake Forest School of Medicine, Department of Family and Community Medicine, Medical Center Boulevard, Winston-Salem, NC 27157 USA; 3 The National Research Center in Complementary and Alternative Medicine (NAFKAM), Department of Community Medicine, UiT The Arctic University of Norway, Sykehusveien 21, 9037 Tromsø, Norway

**Keywords:** Direct risk, Indirect risk, Risk assessment, Patient safety, Conventional health care providers, Complementary therapists, Complementary and alternative medicine: Cancer car

## Abstract

**Background:**

Many complementary therapies offer benefits for patients with cancer. Others may be risky for patients due to negative interactions with conventional treatment and adverse effects. Therefore, cancer patients need guidance from health care providers to assess complementary modalities appropriately to receive benefits and avoid harm.

**Method:**

In a self-administered questionnaire-based cross-sectional study, we compared knowledge and attitudes of health care providers with no training in complementary modalities to that of health care providers with training in complementary modalities about the risks for patients who combine complementary modalities with conventional treatment in cancer care. The analysis was based on responses from 466 participants.

**Results:**

The attitudes and knowledge about direct risk followed provider specialty. Ninety-four percent of the medical doctors, 93% of the nurses, and 87% of the providers with dual training, but 70% of the complementary therapists, believed that complementary modalities can cause adverse effects (*p* < 0.001). The majority of the medical doctors and nurses believed that it is risky to combine complementary and conventional cancer treatments (78% and 93%, respectively), compared to 58% of the providers with dual training and 43% of the complementary therapists (*p* < 0.001). Eighty-nine percent of the medical doctors and nurses believed that complementary modalities should be subjected to more scientific testing before being accepted by conventional health care providers, in contrast to 56% of the dually trained and 57% of the complementary therapists (p < 0.001). The majority of the medical doctors (61%) and nurses (55%) would have neither discouraged nor encouraged the use of complementary modalities if patients asked them for advice. Moreover, less than 1% of the complementary therapists would have discouraged the use of conventional cancer treatments. The study participants believed that the most important factor to recommend a complementary cancer modality to patients is evidence for safety.

**Conclusion:**

The health care providers in this study believed that complementary modalities are associated with direct risk and can cause adverse effects, and that it is risky to combine conventional and complementary treatments due to potential harmful interactions.

## Background

Use of complementary therapies is common among cancer patients both in Norway [1] and worldwide [2, 3]. Complementary therapies are integrated in the care of cancer patients in many countries. In Norway, these modalities are used alongside conventional cancer treatments, mostly practiced outside the conventional health care system and by lay therapists and as complementary to conventional cancer care, rather than as an alternative [4]. The most commonly used complementary modalities in Norway are massage, acupuncture, naprapathy, reflexology, osteopathy, cupping and spiritual healing [5]. Of these therapies, spiritual healing is the complementary therapy most commonly used by cancer patients [6].

Cancer patients often use complementary modalities to relieve pain, and to lessen symptoms of nausea and vomiting associated with chemotherapy or surgical anesthesia, in addition to reduce adverse effects of chemotherapy [2]. One example is using acupuncture to help reduce some adverse effects of cancer treatment [7]. Evidence from randomized controlled trials supports the use of complementary modalities such as acupuncture, massage, music therapy, and relaxation techniques [2, 8]. However, dietary supplements, herbs, and other botanicals can be problematic because of their potential adverse effects or negative interactions with chemotherapy, radiotherapy, or surgery, but may be beneficial when patients do not undergo these treatments [9].

Due to the frequent use of complementary modalities, cancer patients should receive guidance from qualified health care providers about the advantages and disadvantages of complementary modalities in an open, evidence-based, and patient-centered manner [2, 8]. They should be informed about potential benefits, risks, and realistic expectations when combining complementary modalities with conventional treatment in cancer care [10, 11]. However, risks associated with different complementary modalities have been poorly investigated, often due to the assumption that many complementary modalities are “natural” and, therefore, associated with low or no risks.

Risk associated with any health care intervention is generally separated into direct and indirect risk [12]. Direct risk describes the risk associated with an intervention such as harm caused by medical treatments, procedures and pharmacological products. Indirect risk describes a threat to patient safety that is, in the broader sense, associated with the whole treatment setting and clinical practice. Indirect risk may include a provider with limited medical skills who may overlook serious symptoms and thereby cause a delay of necessary conventional treatment [13, 14], or insufficient or inappropriate communication between patients and health care providers with or without complementary training. Another example is continued care in conventional or complementary settings of unproven effectiveness or not conforming to the patients’ values or preferences, while delaying more appropriate complementary modality or conventional care with positive evidence of effectiveness.

One strategy for safely guiding patients in health care decision-making is to examine the risk/benefit ratio; benefits of a health care modality have to clearly outweigh potential risks for providers to consider recommending it to patients. The stronger the evidence for efficacy and safety, the stronger the argument that the modality should be recommended [2]. On the other hand, when the evidence for safety and efficacy is weak, it is sensible to discourage the patients from using the modality. The evidence of safety and efficacy for many complementary modalities is mixed and weak [2, 8], and studies shows that medical doctors find it difficult to recommend many complementary modalities due to a lack of scientific evidence on effect [15–17].

Several complementary therapies offer potential benefits for patients with cancer. Others seem to be ineffective and may present risk for adverse effects or negative interactions. Therefore, it is important for health care providers to guide cancer patients to assess complementary modalities appropriately to receive benefit while avoiding harm.

We investigate what knowledge and attitudes Norwegian health care providers (medical doctors [oncologists and family physicians], nurses, providers with dual training, and complementary therapists [acupuncturists, reflexologists, and massage therapists]) have to guide cancer patients about risks associated with complementary modalities in general when combined with conventional cancer treatment. The aim of this paper is to:

- Compare knowledge and attitudes of health care providers with no training in complementary modalities to that of health care providers with training in complementary modalities about the risks for patients who combine complementary modalities with conventional treatment in cancer care. We consider both direct and indirect risk.

## Method

The main study was conducted from March to June 2016. The study protocol was reviewed and the Regional Committees for Medical and Health Research Ethics (REC) decided that the study did not need REC approval (2012/1318/REK Nord).

### Participants

**Fig. 1:**
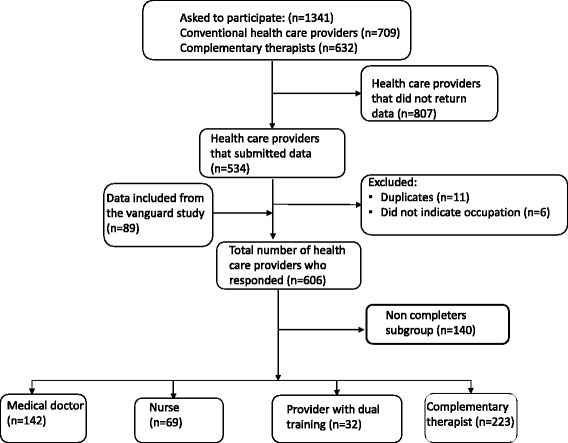
Flow chart of the inclusion process in this study

The study inclusion criteria were being a currently practicing oncology doctor, oncology nurse, family physician, or complementary therapist (acupuncturist, massage therapist, reflexologist/zone-therapist). Participants had to have current or previous clinical experience with cancer patients. All participants were members of a professional association. A total of 1341 participants received an email or a letter with the questionnaire and were asked to participate in the survey. A total of 534 (response rate 40%) completed and returned the questionnaire. We included data from 89 participants from the vanguard study (response rate 24%) [18]. Seventeen participants were excluded because they were duplicates (*n* = 11) or did not indicate a profession (*n* = 6). A total of 140 were non-completers who returned questionnaires that were substantially incomplete; they were excluded from this analysis. The final sample for this analysis included 27 oncologists (18% response rate), 89 nurses (62% response rate) 117 family physicians (25% response rate) and 223 complementary therapists (23% response rate). As several of the participants reported more than one profession, the participants were merged into four mutually exclusive categories: Medical doctors (*n* = 142), nurses (*n* = 69), providers with dual training (*n* = 32), and complementary therapists (*n* = 223) (Fig. 1).

### Data collection

This was a self-administered questionnaire-based cross-sectional study.

#### Questionnaire content

The questionnaire was based on information retrieved from a literature review [17]. The final version included 86 questions in 8 categories (inclusion, communication with patients, risk in clinical practice, perception about complementary and conventional treatment modalities, information gathering about complementary and conventional modalities, personal demographics, and clinical practice or hospital work). Data for this analysis were based on 16 questions about risk in clinical practice presented below.

#### Data collection procedures

Data collection was based on Dillman survey procedures [19]. Through an e-mail, the participants were invited to participate in the study and informed that a new e-mail with a link to the online survey would be sent a week later. One week after the e-mail with the link, a second e-mail was sent as a reminder to the ones who not had responded and as a thank you to the responders. Finally, after an additional week, a reminder repeating the link to the survey was sent to the non-responders. Following the same procedure, the physicians received the questionnaire by post, but with the option to complete the questionnaire by either post or email.

### Measures

#### Professional, personal and clinical practice characteristics

Participants’ professions were reported in the categories medical doctor (oncologists and family physicians not trained and not providing a complementary modality), nurse (oncology nurses not trained and not providing a complementary modality), provider with dual training (a physician or nurse who also provided a complementary modality, or a complementary therapist with conventional training (e.g., physiotherapist), and complementary therapist (including acupuncturists, massage therapists, or reflexologist/zone therapist with no conventional training) (Table 1). Other personal characteristics included age (years), sex (female, male), and level of education (compulsory, middle level, university up to 4 years, university more than 4 years/PhD). Clinical practice characteristics included number of patients and cancer patient visits each week (1–19 patients, 20–39 patients, 40 or more patients), working full time or part time, and the practice/hospital location (rural area, small city or large city).

**Table 1:** Good knowledge of complementary therapies

	Total (*n* = 466)		Medical Doctor (*n* = 142)		Nurse (n = 69)		Provider with dual training (*n* = 32)		Complementary therapist (n = 223)	
	*n*	*%*	*n*	*%*	*n*	*%*	*n*	*%*	*n*	*%*
Acupuncture	233	50.5	27	19	17	25	26	81.3	163	74.4
Homeopathy	73	16.3	18	12.8	1	1.5	2	6.9	52	24.3
Healing	86	18.9	12	8.6	9	13.4	8	26.7	57	26.3
Thai Chi and Chi gong	86	18.8	5	3.5	3	4.4	11	36.7	67	30.7
Aromatherapy	73	15.9	3	2.1	9	13.2	4	13.8	57	25.9
Yoga	136	29.6	27	19	19	28.4	12	40	78	35.3
Mindfullness	163	35.8	41	29.1	22	33.3	13	43.3	87	39.9
Reflexology	101	22	2	1.4	3	4.5	4	12.9	92	41.8
Chinese herbal medicine	36	7.9	2	1.4	1	1.5	6	20.7	27	12.3
Herbal medicine other than Chineese	26	5.8	3	2.2	0	0	1	3.4	22	10.2
Naprapathy	56	12.3	10	7.1	3	4.4	3	10.3	40	18.3
Osteopathy	58	12.7	11	7.9	2	2.9	4	13.3	41	18.7
Massage	219	48.1	34	24.5	19	28.8	19	59.4	147	67.4

#### Direct risk

Five dichotomous measures assess attitudes and knowledge about direct risks of complementary modalities used in cancer treatment: (1) complementary modalities can cause adverse effects; (2) complementary modalities can cause harmful interactions with conventional treatments; (3) treated patients for whom a complementary modality was effective; (4) treated patients for whom a complementary modality was harmful; and (5) combining complementary and conventional treatments increases patient risk.

Since many complementary modalities are practiced outside the established health care service in Norway, we assumed that the knowledge about these modalities was limited among the conventional health care providers. We wanted, therefore,to ask the participants how familiar they were with several complementary modalities, including the following: acupuncture, homeopathy, hands on healing such as Reiki, Tai chi and qigong, aromatherapy, yoga, mindfulness, zone-therapy/reflexology, Chinese herbal medicine, other herbal medicine, chiropractic, osteopathy, and massage. The response options for each of these modalities were: none, little, some, quite a bit and a great deal. In the analysis, these response options were collapsed into “good knowledge” about complementary therapies. A document that explained and defined the different modalities was attached for the participants to use if they were unfamiliar with a specific treatment modality and to ensure that the modalities where understood in the same manner by the participants. The risk question was asked regarding complementary modalities in general and not limited to a specified single therapy (Table 1).

#### Indirect risk

Sixteen measures assess several dimensions of attitudes and knowledge toward indirect risks of complementary modalities used in cancer treatment. (1) Whether complementary modalities should be subjected to more scientific testing is a dichotomous measure. (2) Most important factor for recommending a complementary modality has the values: evidence for safety, evidence for efficacy, evidence does not matter, and double response. (3) Sufficient efficacy evidence to recommend a complementary modality has the values: no or weak evidence, moderate evidence, strong evidence, and would not recommend. (4) Sufficient evidence for safety has the values: no or weak evidence, moderate evidence, strong evidence, and would not recommend.

(5) Number of complementary modality care patients who delayed or declined conventional treatment had the values: none, and one or more. Six dichotomous measures assess how the provider would address a complementary modality patient who delayed or declined conventional treatment: (6) have not experienced; (7) respect patient’s choice; (8) try to convince patient; (9) encourage patient soliciting a second opinion; (10) ask family members to intervene; and (11) inform patient of consequences of not receiving conventional treatment.

(12) Approve of patient combining complementary and conventional treatments has the values: never, sometimes, often, and always. (13) Asking patients if they consider risks of combining complementary and conventional treatments has the values: never, sometimes, and often/always. (14) Advice given to patients who ask about complementary modalities has the values: discourage use, encourage use, neither discourage nor encourage, and other. (15) Advice given to patients who ask about conventional treatment has the values: discourage use, encourage use, neither discourage nor encourage, and other. (16) Complementary modalities are dangerous because they delay conventional treatments is dichotomous.

### Statistical analysis

Descriptive statistics (counts, percentages) were calculated, both for those in each profession and among the four provider groups. Comparisons of provider and practice characteristics across provider types were analyzed. Differences in categorical variables were analyzed using Pearson chi square test or Fisher’s exact test. Analyses of variance were conducted for continuous variables. The significance level was defined as *p* < 0.05 without adjustment for multiple comparisons. The analysis was conducted using SPSSV.24.0 for Windows.

## Results

### Demographics

The professions reported in the study, included oncologists (*n* = 27), family physicians (*n* = 118), oncology nurses (*n* = 89), acupuncturists (*n* = 150), massage therapists (*n* = 82), and reflexologists/zone-therapists (*n* = 35) (Table 1, left column). They were grouped into medical doctor (*n* = 142, 30.5%), nurse (*n* = 69, 14.8%), provider with dual training (*n* = 32, 6.9%) and complementary therapist (*n* = 223, 47.6%) (Table 1, first row).

Medical doctors were significantly younger than the other groups (*p* < 0.001). The groups also differed significantly in education, with medical doctors having the highest proportion with more than 4 years of university training (*p* < 0.001).

Most medical doctors and nurses worked full time (90% and 78%, respectively), compared with complementary therapists (58%) (*p* < 0.001). Medical doctors and providers with dual training reported more patient visits per week (*p* < 0.001). The groups differed in practice locations (*p* = 0.005), with the largest proportion of medical doctors (42%) located in rural areas. The largest proportion of nurses (48%), providers with dual training (48%) and complementary therapists (39%) worked in small towns (Table 1).

### Direct risk

Overall, the attitudes and knowledge about direct risk situations followed the provider profession. Responses were frequently distributed in two opposite ends of the scales. The majority (82%) of the participants agreed that complementary modalities could cause adverse effects. However, discrepancy was found between the different provider groups, as 94% of the medical doctors, 93% of the nurses, and 87% of the providers with dual training, but only 70% of the complementary therapists held this opinion (*p* < 0.001). The contrast was striking between conventional providers and complementary therapists about whether complementary modalities could cause harmful interactions with conventional cancer treatments. Seventy-seven percent of the medical doctors and 85% of the nurses held this opinion, compared to only 52% of the dually trained and 41% of the complementary therapists (*p* < 0.001). Similarly, only 24% of the medical doctors and 49% of the nurses had had cancer patients whose treatment with a complementary modality was effective, in contrast to 96% of the providers with dual training and 86% of the complementary therapists (*p* < 0.001) (Table 2).

**Table 2:** Characteristics of the participants (n = 466)^b^

	Total (n = 466)		Medical doctor (n = 142)		Nurse (n = 69)		Providers with dual training (n = 32)		Complementary therapist (n = 223)		*p*-value
	n	%	n	%	n	%	n	%	n	%	
Gender											< 0.001^*^
Male	108	27.5	69	51.5			3	12.5	36	19.3	
Female	285	72.5	65	48.5	48	100	21	87.5	151	80.7	
Missing	73		8		21		8		36		
Age. years											< 0.001^**^
Mean age	373	48.7	127	45.4	45	51.2	24	52.2	177	50.1	
Missing	93		15		24		8		46		
Education											< 0.001^
Compulsory	2	0.5							2	1.1	
Middle level	33	8.4							33	17.6	
University up to 4 years	112	28.4			23	46.9	11	44	78	41.7	
University more than 4 years/PhD	248	62.8	134	100	26	53.1	14	56	74	39.6	
Missing	71		8		20		7		36		
Profession^*^											
Oncology doctor	27	5.8	27	100							
Family physician	118	25.3	116	99.1			2 ^a^	1.8			
Oncology nurse	89	19.1			69	77.5	20	22.5			
Acupuncturist	150	32.2					25	16.7	125	83.3	
Massage therapist	82	17.6					6	7.3	76	92.7	
Reflexologist/zonetherapist	35	7.5					1	2.9	34	97.1	
Clinical practice											< 0.001^
Full time health provider	287	72.1	121	89.6	38	77.6	18	72	110	58.2	
Part time health provider	92	23.1	11	8.1	10	20.4	5	20	66	34.9	
Other (students or retired persons)	19	4.8	3	2.2	1	2	2	8	13	6.9	
Missing	68		7		20		7		34		
Patient visits per week											< 0.001^*^
1-19 patients	131	33.8	10	7.6	27	57.4	4	16	90	48.6	
20–39 patients	121	31.2	28	21.4	17	36.2	5	20	71	38.4	
40 or more patients	136	35.1	93	71	3	6.4	16	64	24	13	
Missing	78		11		22		7		38		
Cancer patient visits per week											< 0.001^
1–19 cancer patients	361	92.1	125	92.6	31	64.6	23	92	182	98.9	
20 and more patients	31	7.9	10	7.4	17	35.4	2	8	2	1.1	
Missing	74		7		21		7		39		
Location											0.005^*^
Rural area	118	29.7	56	41.5	7	14.6	3	12	52	27.5	
Small city. Village (up to 50.000 inhabitants)	153	38.5	44	32.6	23	47.9	12	48	74	39.2	
Large city (> 50.000 inhabitants)	126	31.7	35	25.9	18	37.5	10	40	63	33.3	
Missing	69		7		21		7		34		

^*^Pearson’s chi-square test; ^**^One way anova test; ^Fisher’s exact test; ^b^Due to multiple response on one or more variables, the analyzed numbers do not always add up to the total number; ^a^These adds to > 32 because providers have more than one area of training

Thirty-eight percent of the medical doctors and 52% of the nurses reported they had ever had cancer patients whose treatment with a complementary modality was harmful, compared to only 12% of the providers with dual training and 9% of the complementary therapies. However, the majority of the participants (75%) responded that they had had *none* or *did not know* of patients whose treatment with a complementary modality was harmful. That is, 62% of the medical doctors, 48% of the nurses, 89% of the dually trained and 91% of the complementary providers (*p* < 0.001). The majority of the medical doctors and nurses believed that it is risky to combine complementary and conventional cancer treatments (78% and 93%, respectively), compared to 58% of the providers with dual training and 43% of the complementary therapists (Table 2).

### Indirect risk

Eighty-nine percent of the medical doctors and nurses believed that complementary modalities should be subjected to more scientific testing before being accepted by conventional health care providers, in contrast to 56% of the dually trained and 57% of the complementary therapists (p < 0.001). Overall, the participants believed that evidence for safety was more important than evidence for efficacy to recommend a complementary modality to cancer patients (49% and 40%, respectively). However, a significant difference was found among the provider groups. Thirty-eight percent of the medical doctors and 44% of the providers with dual training believed that evidence for *safety* was the most important, compared to 60% of the nurses, and 54% of the complementary therapists. Less than half of the medical doctors, nurses and complementary therapists believed that evidence for *efficacy* was the most important to recommend a complementary modality, in contrast to 52% of the dually trained (*p* < 0.001). Four percent of the medical doctors and 9% of the complementary therapists believed that *evidence did not matter* (< 0.001) (Table 3).

**Table 3:** Attitudes and knowledge about direct risk situations (n = 466)^a^

	Total		Medical doctor (n = 142)		Nurse (n = 69)		Provider with dual training n = 32)		Complementary therapist (n = 223)		p-value
	*n*	*%*	*n*	*%*	*n*	*%*	*n*	*%*	*n*	*%*	
Complementary modalities can cause adverse effects^^											< 0.001^*^
Yes	376	82.1	134	94.4	64	92.8	27	87.1	151	69.9	
Complementary modalities can cause harmful interactions with conventional treatments											< 0.001^*^
Yes	265	59.2	105	76.6	55	84.6	16	51.6	89	41.4	
Treated patiens for whom a complementary modality was effective											< 0.001^*^
Yes	239	60.8	32	24.2	27	49.1	25	96.2	155	86.1	
Treated patients for whom a complementary modality was harmful											< 0.001^*^
Yes	97	24.7	51	38.3	27	51.9	3	11.5	16	8.8	
Combining complementary and conventional treatments increases patient risk											< 0.001^*^
Yes	240	62.8	101	77.7	49	92.5	14	58.3	76	43.4	

^*^Pearson’s chi-square test; ^Fisher’s exact test; ^a^Due to multiple and missing responses, the analyzed numbers do not always add up to the total number;^^The missing response variated between (*n* = 8 and *n* = 84)

The contrast was striking between conventional health care providers and complementary therapists in what constitutes enough evidence on efficacy to recommend a complementary modality to cancer patients. The providers with dual training and complementary therapists believed that *moderate evidence for efficacy* was enough (63% and 44%, respectively), while only 27% of the medical doctors and 18% of the nurses held this opinion. On the other hand, 49% of the medical doctors and 64% of the nurses believed that only *strong evidence for efficacy* was enough, compared to 26% of the providers with dual training and 29% of the complementary therapists. Eighteen percent of the medical doctors, 13% of the nurses and 14% of the complementary therapists *would never recommend* a complementary modality because of efficacy concerns, 4% of the providers with dual training agreed (*p* < 0.001) (Table 3).

Overall, 74% of the participants believed that *strong evidence for safety* was the most important to recommend a complementary modality to cancer patients. Eighty percent of the medical doctors and 84% of the nurses held this belief, in contrast to 67% of the providers with dual training and complementary therapists. Less than 15% of the medical doctors and nurses *would never recommend* complementary therapy because of safety concerns, compared to 4% of the providers with dual training and 7% of the complementary therapists (*p* < 0.001).

Almost all of the providers with dual training and complementary therapists had no patients who delayed or declined conventional cancer treatment during the past year (96% and 94%, respectively), compared to 70% of the medical doctors and 49% of the nurses. Conversely, 30% of the medical doctors and 51% of the nurses had one or more patients who delayed or declined conventional cancer treatment during the past year, in contrast to 4% of the dually trained and 6% of the complementary therapists (*p* < 0.001).

Between group differences were found regarding how to handle cancer patients who choose to decline or delay conventional cancer treatment. Most of the medical doctors (63%), nurses (78%) and providers with dual training (61%) had experienced this, compared to 41% of the complementary therapists (p < 0.001). Forty percent of the medical doctors and 51% of the nurses would respect patient’s choice, in contrast to 36% of the providers with dual training, and 25% of the complementary therapists (p < 0.001). Moreover, 46% of the medical doctors would try to convince patient to consider conventional treatment, compared to 19% of the nurses, 23% of the providers with dual training, and 19% of the complementary therapists. Fewer between-group differences were found in encouraging the patient soliciting a second opinion, where less than 40% of each groups would have encouraged patients to do so (*p* = 0.122). Less than 5% of medical doctors and nurses would ask family members to intervene; however, none of the providers with dual training or complementary therapists would do so (*p* = 0.003). More than 60% of the medical doctors and nurses would have informed the patient of consequences of not receiving conventional treatment, in contrast to 32% of the providers with dual training and 28% of the complementary therapists (< 0.001) (Table 3).

Fewer between-group differences were found regarding approval of cancer patients´ use of complementary therapy combined with conventional treatment. Less than 10% of each provider group would *never* approve such use. However, 63% of the medical doctors and 60% of the nurses would *sometimes* approve such use, compared to 24% of the providers with dual training and 15% of the complementary therapists. About than 35% of the providers with dual training and complementary therapists would *often* approve such use, compared to the medical doctors and nurses (20% and 17%, respectively). Eight percent of the medical doctors and 17% of the nurses would *always* approve the use of complementary therapy, in contrast to 44% of the providers with dual training and 46% of the complementary therapists (*p* < 0.001).

Less than half of the medical doctors, nurses, providers with dual training and complementary therapists reported that they had *never* asked their patients if they considered the risk of combining complementary with conventional treatment. Between 44% and 55% of medical doctors, nurses and providers with dual training answered that they *sometimes* asked about this. This was in contrast to the complementary therapists that *often/always* asked patients about perceived risk of combining complementary and conventional treatment (33%) (p < 0.001).

A significant difference was found between the provider groups regarding what advice to give to cancer patients if they asked about complementary therapy. Eight percent of the medical doctors and 4% of the nurses would *discourage* the use of complementary modalities, compared to none of the dually trained and 1% of the complementary therapists. Two percent of the medical doctors and 7% of the nurses would *encourage* the use, in contrast to 42% of the dually trained and 47% of the complementary therapists. The majority of the medical doctors (61%) and nurses (55%) would have done *neither*, compared to providers with dual training (39%) and complementary therapists (32%) (Table 3).

Less than 1% of each provider group would have discouraged the use of conventional medicine if patients asked for advice. The majority of the participants (78%) would have encouraged the use of conventional cancer treatment. However, a significant difference was found among provider groups. Eighty-seven percent of the medical doctors, 78% of the nurses and 84% of the dually trained encouraged such use, in contrast to 71% of the complementary therapists (*p* = 0.005).

Seventy-eight percent of the medical doctors and 71% of the nurses believed complementary therapy could be dangerous because it may delay conventional treatment, compared to 24% of the providers with dual training and 22% of the complementary therapists (*p* < 0.001) (Table 3), (Table 4).

**Table 4:** Attitudes and knowledge about indirect risk situations (*n* = 466)^a^

	Total		Medical doctor (n = 142)		Nurse (n = 69)		Provider with dual training (n = 32)		Complementary therapist (n = 223)		p-value
	*n*	*%*	*n*	*%*	*n*	*%*	*n*	*%*	*n*	*%*	
Whether complementary modalities should be subjected to more scientific testing^^											
Yes	302	71.6	119	88.8	54	88.5	15	55.6	114	57	< 0.001^*^
Most important factor for recommending a complementary modality											< 0.001^
Evidence for safety	209	49.1	52	38.2	37	59.7	12	44.4	108	53.7	
Evidence for efficacy	169	39.7	55	40.4	25	40.3	14	51.9	75	37.3	
Evidence does not matter	22	5.2	5	3.7	0	0	0	0	17	8.5	
Double responses	26	6.1	24	17.6	0	0	1	3.7	1	0.5	
Sufficient efficacy evidence to recommend a complementary modality											< 0.001^
No or weak evidence for efficacy	39	9.3	9	6.6	3	4.9	2	7.4	25	12.9	
Moderate evidence for efficacy	151	36.1	37	27.2	11	18	17	63	86	44.3	
Strong evidence for efficacy	168	40.2	66	48.5	39	63.9	7	25.9	56	28.9	
Would never recommend	60	14.4	24	17.6	8	13.1	1	3.7	27	13.9	
Sufficient evidece for safety											< 0.001^
No or weak evidence for safety	12	2.9	3	2.2	0	0	1	3.7	8	4.1	
Moderate evidence for safety	57	13.6	4	3	3	4.8	7	25.9	43	22.1	
Strong evidence for safety	309	73.7	108	80	52	83.9	18	66.7	131	67.2	
Would never recommend	41	9.8	20	14.8	7	11.3	1	3.7	13	6.7	
Number of complementary modality care patients who delayed or declined conventional treatment											< 0.001^*^
No patients	337	80	95	70.4	30	49.2	25	96.2	187	94	
One or more patients	84	20	40	29.6	31	50.8	1	3.8	12	6	
How the providers would address a complementary modality patient who delayed or declined conventional treatment											
Have not experienced	206	44.2	52	36.6	15	21.7	12	38.7	127	58.8	< 0.001^*^
Respect the patient’s choice	157	33.7	57	40.1	35	50.7	11	35.5	54	25.0	< 0.001^*^
Try to convince patient	127	27.3	65	45.8	13	18.8	7	22.6	42	19.4	< 0.001^*^
Encourage patient soliciting a second opinion	150	32.2	34	23.9	24	34.8	12	38.7	80	37.0	0.122^*^
Ask family members to intervene	9	1.9	7	4.9	2	2.9	0	0.0	0	0.0	0.003^
Inform patient of consequences of not receiving conventional treatment	203	43.6	91	64.1	42	60.9	10	32.3	60	27.8	< 0.001^*^
Approve of patient combining complementary and conventional treatments											< 0.001^
Never	23	5.9	12	9	3	5.8	0	0	8	4.5	
Sometimes	147	37.8	84	63.2	31	59.6	6	24	26	14.5	
Often	107	27.5	27	20.3	9	17.3	8	32	63	35.2	
Always	112	28.8	10	7.5	9	17.3	11	44	82	45.8	
Asking patients if they consider risks of combining complementary and conventional treatments											< 0.001^*^
Never	153	39.2	54	40.3	17	30.4	11	44	71	40.6	
Sometimes	147	37.7	59	44	31	55.4	11	44	46	26.3	
Often/always	90	23.1	21	15.7	8	14.3	3	12	58	33.1	
Advice given to patients who ask about complementary modalities											< 0.001^
Discourage use	14	3.6	10	7.5	2	3.6	0	0	2	1.1	
Encourage use	100	25.8	3	2.3	4	7.3	11	42.3	82	47.1	
Neither	177	45.6	81	60.9	30	54.5	10	38.5	56	32.2	
Other	97	25	39	29.3	19	34.5	5	19.2	34	19.5	
Advice given to patients who ask about conventional treatment											
Discourage use	2	0.5	0	0	0	0	0	0	2	1.1	0.005^
Encourage use	308	78.2	116	86.6	42	77.8	21	84	129	71.3	
Neither discourage nor encourage	46	11.7	5	3.7	6	11.1	4	16	31	17.1	
Other	38	9.6	13	9.7	6	11.1	0	0	19	10.5	
Complementary modalities are dangerous because they delay conventional treatments											< 0.001^*^
Yes	178	47.6	100	78.1	35	71.4	6	24	37	21.5	

^*^Pearson’s chi-square test; ^Fisher’s exact test; ^a^Due to multiple and missing responses, the analyzed numbers do not always add up to the total number;^^The missing response variated between (n = 8 and n = 84)

## Discussion

Generally, the attitudes and knowledge about complementary therapies in cancer care followed provider specialty. The conventional and complementary providers were mostly in favor of their own discipline. The majority of the health care providers in this study believed that complementary modalities could cause adverse effects and harmful interactions with conventional cancer treatment. This is in line with Hyodo et al. [20] who investigated the attitudes of Japanese clinical oncologists towards complementary cancer modalities (herbs and over the counter products); a total of 84% of the oncologists considered the possibility of drug interactions between anticancer drugs and these products. As regards harmful interactions, more than half of the complementary therapists in our study did not support this view.

Two-thirds of the medical doctors and more than three-quarters of the nurses believed that it could be risky for patients to combine complementary and conventional cancer treatments. In addition, they believed that using complementary modalities could be risky because it might delay conventional treatment. The complementary therapists believed that to a lesser extent. However, the assumption that risk associated with many complementary modalities is indirect is in accordance with Fisher [13], Dantas [21], and Stub [17]. They found that the main risks associated with homeopathy and other complementary modalities were indirect; that is, related to the therapists and clinical practice rather than the medicine.

Moreover, the conventional health care providers claimed that complementary modalities should be subjected to more scientific testing, and that evidence for safety was equally important as evidence for efficacy. To recommend a complementary modality, they believed that strong evidence for safety was most important. This is in line with Fønnebø et al. [22]. His research group recommended a new strategy for assessing complementary therapies because many of these modalities have no regulatory or financial gatekeepers controlling their therapeutic tools/agents prior to marketing. Consequentially, many modalities are in widespread use before researchers know of their existence and prior to any consideration about safety issues. The group proposed a five-phased research strategy, where safety status should be assessed before the assessment of efficacy.

If patients asked them for advice, the majority (78%) of the participants in the study and 71% of the complementary therapists would encourage the use of conventional cancer treatment. Only two complementary therapists (1%) would discourage the use of conventional medicine. These results are in contrast to Cassileth [23] who conducted a survey among patients and complementary therapists in the USA, and found that 22% of complementary therapists were supportive of cancer patient’s use of conventional care, 36% were neutral and 21% disparaged conventional medicine. However, research has revealed that the reasons for cancer patient to decline or delay conventional treatment may be due to the medical doctors’ communication style. In a qualitative study, Salamonsen [24] investigated a possible relationship between cancer patients’ communication experiences with doctors and the decision to use complementary modalities as either a supplement or alternative to conventional treatment. She found three doctor communication styles that influenced the patients’ treatment decisions: (i) negative communication experiences because of the use of complementary modalities; (ii) negative communication experiences resulted in the decision to use complementary modalities, and in some cases to decline conventional treatment; and (iii) positive communication experiences that led to the decision to use complementary modalities as a supplement, and not as an alternative to conventional treatment.

Cancer patients who decline or delay conventional cancer treatment should, according to two-thirds of the medical doctors, be informed about the consequences of not applying conventional treatment. More than half of the nurses strongly believed in respecting the patient’s autonomy; they believed this to a greater degree than did complementary therapists. This is the opposite of what one would expect, but might be explained by the nurses’ strong tradition of being the patients’ advocates [15]. Moreover, more than half of the medical doctors and nurses neither discouraged nor encouraged the use of complementary modalities. Advice may depend on the situation, the medical doctor’s knowledge of the complementary therapy and cancer type [17].

### Practical implementation

Many complementary therapies offer potential benefits for patients with cancer. Others seem to be ineffective and may present risks of adverse effects or interactions. Therefore, it is important for health care providers to guide cancer patients to assess complementary modalities appropriately to receive benefit while avoiding harm. In the training of doctors and nurses, a greater awareness of potential positive and negative outcomes of combining conventional and complementary treatment should be emphasized. More research that investigates direct and indirect risks of combining these treatment modalities in cancer care is warranted.

### Limitations

The main limitation of this study is the rather low response rate among the physicians and the complementary therapists. With a 25% response rate or lower in these groups, the findings are only the expression of a highly selected group of physicians and complementary therapists. We might therefore conservatively conclude that the findings of this study are valid for 18–25% of the physicians and complementary therapists, as the non-respondents may differ significantly from those who responded [25]. We presume that physicians with interest in complementary therapies might be over-represented, as interest in the topic studied has shown to increase the response rate [26]. However, our findings are similar to those reported by Hyodo and Frenkel [20, 27]. This suggests that non-response bias poses a limited threat to our study’s validity [28]. When it comes to nurses, however, the findings are more generalizable as the response rate was much higher, 62%. The high number of nurses (*n* = 20) who had additional education as a complementary therapist indicates, however, that also among nurses, therapists with an interest for complementary therapies might be over-represented.

## Conclusion

The health care providers in this study believed that complementary modalities used in cancer care are associated with direct risk and can cause adverse effects, mostly due to harmful interactions. However, the majority of the complementary therapists in this study encourage their patients to apply conventional cancer treatment. The most important factor to recommend a complementary cancer modality to patients was found to be evidence for safety.
